# A radiomics-based comparative study on arterial spin labeling and dynamic susceptibility contrast perfusion-weighted imaging in gliomas

**DOI:** 10.1038/s41598-020-62658-9

**Published:** 2020-04-09

**Authors:** Takashi Hashido, Shigeyoshi Saito, Takayuki Ishida

**Affiliations:** 1 Department of Medical Technology, Osaka University Hospital, Suita, Osaka Japan; 2 Department of Medical Physics and Engineering, Division of Health Science, Osaka University Graduate School of Medicine, Suita, Osaka Japan

**Keywords:** CNS cancer, Tumour heterogeneity, Magnetic resonance imaging

## Abstract

Radiomics has potential for reflecting the differences in glioma perfusion heterogeneity between arterial spin labeling (ASL) and dynamic susceptibility contrast (DSC) imaging. The aim of this study was to compare radiomic features of ASL and DSC imaging-derived parameters (cerebral blood flow, CBF) and assess radiomics-based classification models for low-grade gliomas (LGGs) and high-grade gliomas (HGGs) using their parameters. The ASL-CBF and DSC-relative CBF of 46 glioma patients were normalized (ASL-nCBF and DSC-nrCBF) for data analysis. For each map, 91 radiomic features were extracted from the tumor volume. Seventy-five radiomic features were significantly different (*P* < 0.00055) between ASL-nCBF and DSC-nrCBF. Positive correlations were observed in 75 radiomic features between ASL-nCBF and DSC-nrCBF. Even though ASL imaging underestimated CBF compared with DSC imaging, there were significant correlations (*P* < 0.00055) in the first-order-based mean, median, 90^th^ percentile, and maximum. Texture analysis showed that ASL-nCBF and DSC-nrCBF characterized similar perfusion patterns, while ASL-nCBF could evaluate perfusion heterogeneity better. The areas under the curve of the ASL-nCBF and DSC-nrCBF radiomics-based classification models for gliomas were 0.888 and 0.962, respectively. Radiomics in ASL and DSC imaging is useful for characterizing glioma perfusion patterns quantitatively and for classifying LGGs and HGGs.

## Introduction

Angiogenesis, the formation of new blood vessels for tumor growth, plays a key role in gliomas^1^. Gliomas rely on angiogenesis to maintain an adequate blood supply for the delivery of nutrients and oxygen, thereby consisting of a complex and heterogeneous vasculature^1,2^. Therefore, high-grade gliomas (HGGs) are characterized by heterogeneous and relatively higher perfusion compared with low-grade gliomas (LGGs)^3^. These characteristics are critical elements in the determination of preoperative grade, treatment strategy, and prognosis of gliomas.

Magnetic resonance (MR) perfusion-weighted imaging can provide information about tumor vascularity. Cerebral blood flow (CBF) and cerebral blood volume (CBV) are representative perfusion parameters that can be measured by MR perfusion-weighted imaging. Dynamic susceptibility contrast (DSC) imaging is an established MR perfusion-weighted imaging^4^. DSC imaging requires a bolus injection of a gadolinium-based (exogenous) contrast agent. A deconvolution method is used for the quantification analysis of relative CBF (rCBF) and mean transit time (MTT)^4^. Relative CBV (rCBV) is related to rCBF and MTT via the equation MTT = rCBV/rCBF, and can be estimated from the area of the time–concentration curve in tissue^4^. DSC imaging-derived parameters (rCBF and rCBV) are known to correlate with histological findings of glioma angiogenesis^5,6^ and glioma grade^7–9^.

On the other hand, arterial spin labeling (ASL) imaging, which is another MR perfusion-weighted imaging, does not require an exogenous contrast agent^10^. ASL imaging allows absolute quantification of CBF by using magnetically labeled arterial blood water as an endogenous tracer^10^. Some studies have compared ASL with DSC imaging in brain tumors^11–14^. These studies concluded that ASL imaging can be an alternative method to DSC imaging for evaluating brain tumors. The usefulness of ASL imaging for differentiation between LGGs and HGGs has also been reported^15–18^. Thus, ASL imaging is establishing itself as a non-invasive perfusion measurement without a contrast agent and X-ray exposure, making it valuable for renal failure^19^ and pediatric patients^20^.

Radiomics can describe tumor phenotypic characteristics using various quantitative features based on histogram and texture in medical images^21,22^. Recently, several studies have reported that radiomic features in MR imaging reflect tumor heterogeneity and have potential for predicting glioma grading^23,24^. We assumed that radiomics has the potential for quantitative assessment of glioma perfusion heterogeneity in ASL and DSC imaging. However, because ASL and DSC imaging are based on different techniques, it is unclear whether they exhibit similar perfusion patterns (e.g., heterogeneity) or how their perfusion patterns differ. In order to use ASL imaging as an alternative to DSC imaging, it is necessary to investigate this issue. Therefore, the purpose of this study was to compare radiomic features of ASL and DSC imaging in gliomas. Additionally, we constructed radiomics-based classification models for glioma grading using ASL and DSC imaging and assessed their diagnostic performance.

## Results

### Glioma patients

**Figure 1:**
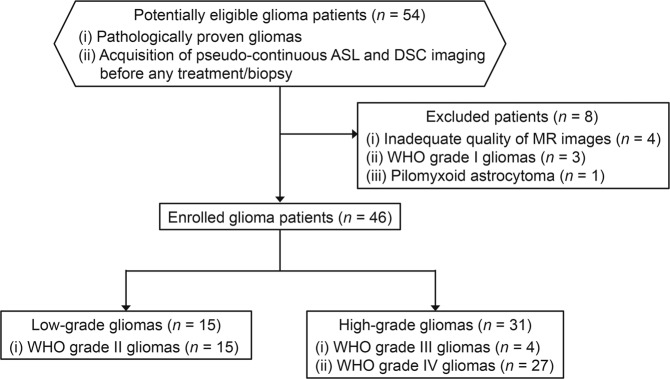
Flow diagram for glioma patient selection. ASL, arterial spin labeling; DSC, dynamic susceptibility contrast; MR, magnetic resonance; WHO, World Health Organization.

Figure 1 shows the flow diagram for glioma patient selection in this study. Among the 46 gliomas enrolled, 15 gliomas were LGGs (World Health Organization (WHO) grade II; 11 males and 4 females; mean age, 41.5 ± 12.6 years), and 31 gliomas were HGGs (WHO grade III/IV; 25 males and 6 females; mean age, 57.7 ± 16.7 years). All LGGs were WHO grade II (diffuse astrocytoma = 11, oligodendroglioma = 4). The HGGs included were WHO grade III (anaplastic astrocytoma = 3, anaplastic oligodendroglioma = 1) and IV (glioblastoma = 26, diffuse midline glioma = 1).

### Comparisons of radiomic features between ASL and DSC imaging

**Figure 2:**
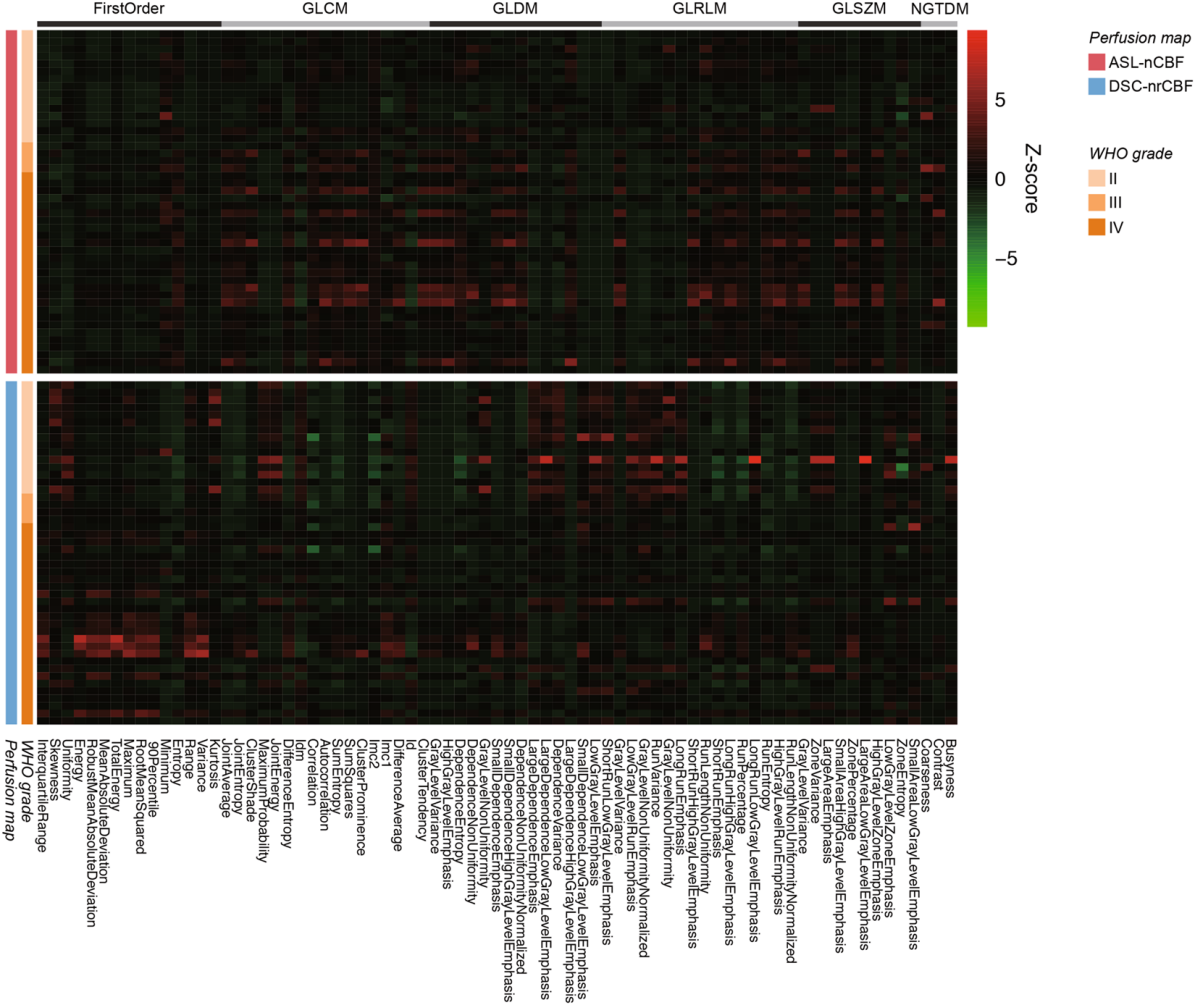
Heatmap of radiomic features for comparing between ASL-nCBF and DSC-nrCBF. All the 75 radiomic features shown in the heatmap were found to be significantly different (*P* < 0.00055) between ASL-nCBF and DSC-nrCBF. Rows correspond to patients, and columns correspond to Z-score normalized radiomic features. The heatmap is grouped by perfusion map, glioma grade, and radiomic feature class. ASL-nCBF, arterial spin labeling normalized cerebral blood flow; DSC-nrCBF, dynamic susceptibility contrast normalized relative cerebral blood flow; WHO, World Health Organization; GLCM, gray-level co-occurrence matrix; GLDM, gray-level dependence matrix; GLRLM, gray-level run-length matrix; GLSZM, gray-level size-zone matrix; NGTDM, neighboring gray-tone difference matrix.

All 91 radiomic features (Supplementary Table S1) in all gliomas were compared between ASL-normalized CBF (ASL-nCBF) and DSC-normalized rCBF (DSC-nrCBF) using the paired *t*-test or Wilcoxon signed-rank test as appropriate. Figure 2 shows the heatmap of the 75 radiomic features that were significantly different (*P* < 0.00055). As shown in Fig. 2 and Supplementary Table S2, 15 out of the 18 first-order-based features (83.3%), including the minimum (ASL-nCBF, 0.49 ± 0.31; DSC-nrCBF, 0.13 ± 0.26), 90^th^ percentile (ASL-nCBF, 3.15 ± 1.51; DSC-nrCBF, 5.64 ± 4.75), and maximum (ASL-nCBF, 5.30 ± 2.83; DSC-nrCBF, 24.41 ± 21.77), had significant differences (*P* < 0.00055) between ASL-nCBF and DSC-nrCBF. Sixty out of the 73 texture features (82.2%), including features from all 5 texture classes, had significant differences (*P* < 0.00055) between ASL-nCBF and DSC-nrCBF. The mean and standard deviation of all radiomic features and statistical results are shown in Supplementary Table S2.

### Correlations of radiomic features between ASL and DSC imaging

**Figure 3:**
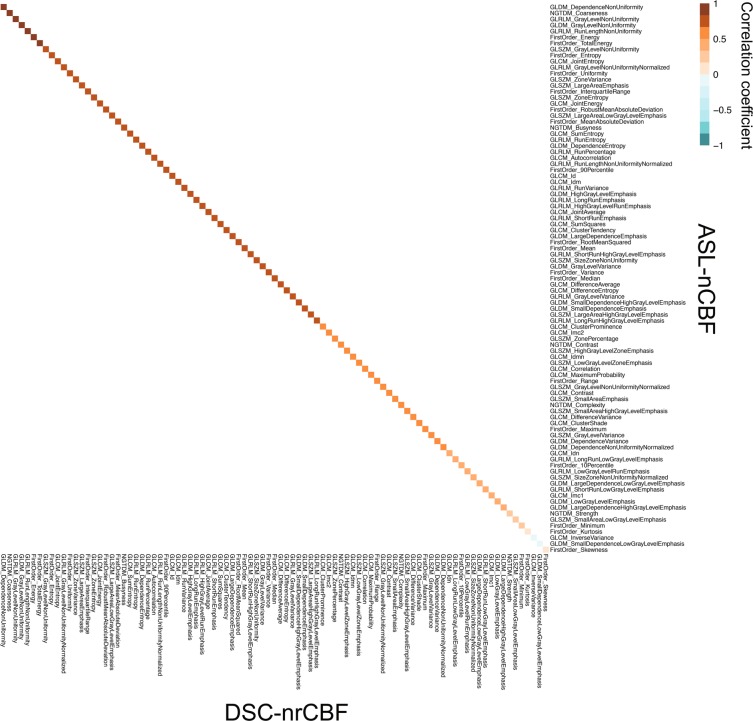
Heatmap of correlation coefficients between ASL-nCBF and DSC-nrCBF radiomic features. Brown and blue colors represent positive and negative correlations, respectively. The correlation coefficients are arranged in order from the highest significant correlation in the upper left corner, and the corresponding features are shown in the x- and y-axes. Significant correlations (*P* < 0.00055) were observed in the top 75 radiomic features. ASL-nCBF, arterial spin labeling normalized cerebral blood flow; DSC-nrCBF, dynamic susceptibility contrast normalized relative cerebral blood flow; GLCM, gray-level co-occurrence matrix; GLDM, gray-level dependence matrix; GLRLM, gray-level run-length matrix; GLSZM, gray-level size-zone matrix; NGTDM, neighboring gray-tone difference matrix.

Figure 3 shows the heatmap of Pearson’s product-moment correlation coefficients (*r*) or Spearman’s rank-order correlation coefficients (*ρ*) of all radiomic features between ASL-nCBF and DSC-nrCBF in all gliomas. Significant correlations (*P* < 0.00055) were observed in 14 first-order-based features (14/18, 77.8%) and 61 texture features (61/73, 83.6%). The detailed results of the correlation analysis can be found in Supplementary Table S3.

### Radiomics-based classification models for LGGs and HGGs

Logistic regression models were constructed using the radiomic features selected by least absolute shrinkage and selection operator (LASSO) and Eq. (3) in the Materials and methods section, as follows:

1 $$\begin{array}{ccl}{\rm{l}}{\rm{o}}{\rm{g}}{\rm{i}}{\rm{t}}(p{)}_{{\rm{A}}{\rm{S}}{\rm{L}}\text{-}{\rm{n}}{\rm{C}}{\rm{B}}{\rm{F}}} & = & 1.53-2.67\times {\rm{G}}{\rm{L}}{\rm{C}}{\rm{M}}\,{\rm{i}}{\rm{n}}{\rm{v}}{\rm{e}}{\rm{r}}{\rm{s}}{\rm{e}}\,{\rm{v}}{\rm{a}}{\rm{r}}{\rm{i}}{\rm{a}}{\rm{n}}{\rm{c}}{\rm{e}}\\  &  & -\,1.14\times {10}^{-4}\times \,{\rm{G}}{\rm{L}}{\rm{R}}{\rm{L}}{\rm{M}}\,{\rm{g}}{\rm{r}}{\rm{a}}{\rm{y}}\text{-}{\rm{l}}{\rm{e}}{\rm{v}}{\rm{e}}{\rm{l}}\,{\rm{n}}{\rm{o}}{\rm{n}}\text{-}{\rm{u}}{\rm{n}}{\rm{i}}{\rm{f}}{\rm{o}}{\rm{r}}{\rm{m}}{\rm{i}}{\rm{t}}{\rm{y}}\\  &  & -\,1.37\times {10}^{1}\times \,{\rm{G}}{\rm{L}}{\rm{S}}{\rm{Z}}{\rm{M}}\,{\rm{g}}{\rm{r}}{\rm{a}}{\rm{y}}\text{-}{\rm{l}}{\rm{e}}{\rm{v}}{\rm{e}}{\rm{l}}\,{\rm{n}}{\rm{o}}{\rm{n}}\text{-}{\rm{u}}{\rm{n}}{\rm{i}}{\rm{f}}{\rm{o}}{\rm{r}}{\rm{m}}{\rm{i}}{\rm{t}}{\rm{y}}\,{\rm{n}}{\rm{o}}{\rm{r}}{\rm{m}}{\rm{a}}{\rm{l}}{\rm{i}}{\rm{z}}{\rm{e}}{\rm{d}}\\  &  & +\,5.18\times \,{\rm{G}}{\rm{L}}{\rm{S}}{\rm{Z}}{\rm{M}}\,{\rm{s}}{\rm{i}}{\rm{z}}{\rm{e}}\text{-}{\rm{z}}{\rm{o}}{\rm{n}}{\rm{e}}\,{\rm{n}}{\rm{o}}{\rm{n}}\text{-}{\rm{u}}{\rm{n}}{\rm{i}}{\rm{f}}{\rm{o}}{\rm{r}}{\rm{m}}{\rm{i}}{\rm{t}}{\rm{y}}\,{\rm{n}}{\rm{o}}{\rm{r}}{\rm{m}}{\rm{a}}{\rm{l}}{\rm{i}}{\rm{z}}{\rm{e}}{\rm{d}}\\  &  & -\,1.59\times {10}^{1}\times \,{\rm{G}}{\rm{L}}{\rm{S}}{\rm{Z}}{\rm{M}}\,{\rm{s}}{\rm{m}}{\rm{a}}{\rm{l}}{\rm{l}}\,{\rm{a}}{\rm{r}}{\rm{e}}{\rm{a}}\,{\rm{l}}{\rm{o}}{\rm{w}}\,{\rm{g}}{\rm{r}}{\rm{a}}{\rm{y}}\text{-}{\rm{l}}{\rm{e}}{\rm{v}}{\rm{e}}{\rm{l}}\,{\rm{e}}{\rm{m}}{\rm{p}}{\rm{h}}{\rm{a}}{\rm{s}}{\rm{i}}{\rm{s}}\end{array}$$

2 $$\begin{array}{ccl}{\rm{l}}{\rm{o}}{\rm{g}}{\rm{i}}{\rm{t}}(p{)}_{{\rm{D}}{\rm{S}}{\rm{C}}\text{-}{\rm{n}}{\rm{r}}{\rm{C}}{\rm{B}}{\rm{F}}} & = & 2.13\times {10}^{-1}-1.08\times {10}^{-5}\times \,{\rm{G}}{\rm{L}}{\rm{D}}{\rm{M}}\,{\rm{g}}{\rm{r}}{\rm{a}}{\rm{y}}\text{-}{\rm{l}}{\rm{e}}{\rm{v}}{\rm{e}}{\rm{l}}\,{\rm{n}}{\rm{o}}{\rm{n}}\text{-}{\rm{u}}{\rm{n}}{\rm{i}}{\rm{f}}{\rm{o}}{\rm{r}}{\rm{m}}{\rm{i}}{\rm{t}}{\rm{y}}\\  &  & -\,1.26\times {10}^{-1}\times \,{\rm{G}}{\rm{L}}{\rm{D}}{\rm{M}}\,{\rm{d}}{\rm{e}}{\rm{p}}{\rm{e}}{\rm{n}}{\rm{d}}{\rm{e}}{\rm{n}}{\rm{c}}{\rm{e}}\,{\rm{v}}{\rm{a}}{\rm{r}}{\rm{i}}{\rm{a}}{\rm{n}}{\rm{c}}{\rm{e}}\\  &  & -\,1.77\times {\rm{f}}{\rm{i}}{\rm{r}}{\rm{s}}{\rm{t}}\text{-}{\rm{o}}{\rm{r}}{\rm{d}}{\rm{e}}{\rm{r}}\,{\rm{m}}{\rm{i}}{\rm{n}}{\rm{i}}{\rm{m}}{\rm{u}}{\rm{m}}\\  &  & -\,7.90\times {10}^{-3}\times \,{\rm{f}}{\rm{i}}{\rm{r}}{\rm{s}}{\rm{t}}\text{-}{\rm{o}}{\rm{r}}{\rm{d}}{\rm{e}}{\rm{r}}\,{\rm{k}}{\rm{u}}{\rm{r}}{\rm{t}}{\rm{o}}{\rm{s}}{\rm{i}}{\rm{s}}\\  &  & -\,2.49\times {10}^{-2}\times {\rm{G}}{\rm{L}}{\rm{R}}{\rm{L}}{\rm{M}}\,{\rm{r}}{\rm{u}}{\rm{n}}\,{\rm{v}}{\rm{a}}{\rm{r}}{\rm{i}}{\rm{a}}{\rm{n}}{\rm{c}}{\rm{e}}\\  &  & +\,7.12\times {10}^{-1}\times \,{\rm{G}}{\rm{L}}{\rm{S}}{\rm{Z}}{\rm{M}}\,{\rm{z}}{\rm{o}}{\rm{n}}{\rm{e}}\,{\rm{e}}{\rm{n}}{\rm{t}}{\rm{r}}{\rm{o}}{\rm{p}}{\rm{y}}\end{array}$$

where GLCM, GLDM, GLRLM, and GLSZM are the gray-level co-occurrence matrix, gray-level dependence matrix, gray-level run-length matrix, and gray-level size-zone matrix, respectively.

**Figure 4:**
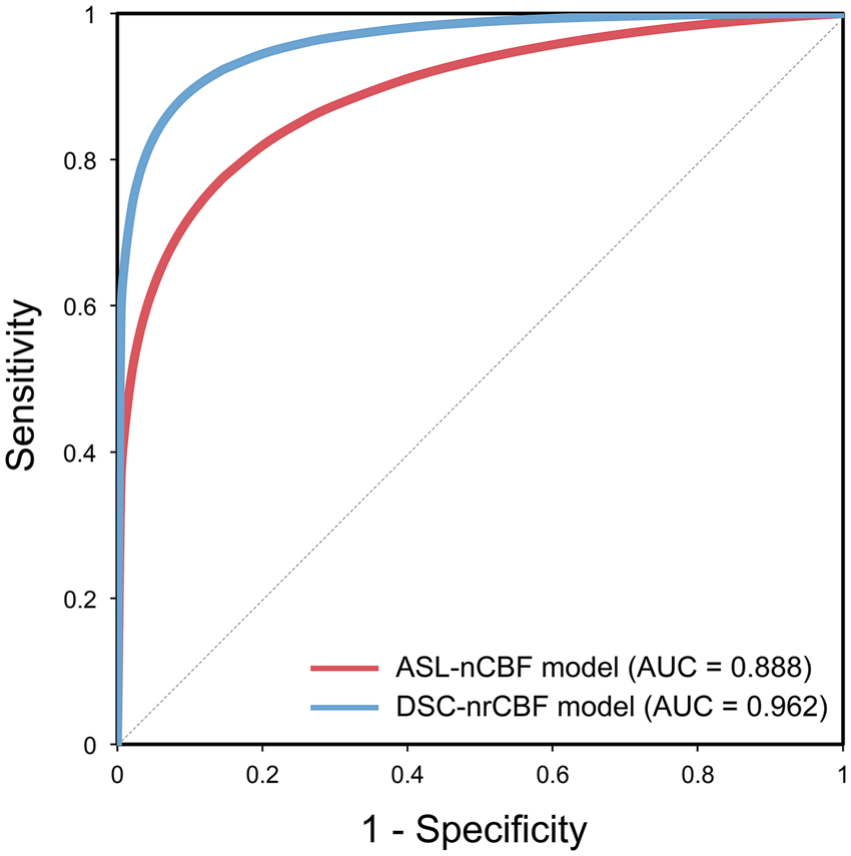
Receiver operating characteristic curves for the radiomics-based classification models for low- and high-grade gliomas. The red and blue solid lines represent the curves of the ASL-nCBF and DSC-nrCBF models, respectively. ASL-nCBF, arterial spin labeling normalized cerebral blood flow; DSC-nrCBF, dynamic susceptibility contrast normalized relative cerebral blood flow; AUC, area under the curve.

According to the receiver operating characteristic (ROC) analysis (Fig. 4 and Table 1), the DSC-nrCBF model (AUC, 0.962; sensitivity, 89.3%; specificity, 92.9%) showed better diagnostic performance for differentiating HGGs from LGGs than the ASL-nCBF model (AUC, 0.888; sensitivity, 85.7%; specificity, 85.7%). However, no significant differences (*P* = 0.133) were found between the areas under the curve (AUCs) of the models.

**Table 1:** Receiver operating characteristic analysis results of radiomics-based classification models for low- and high-grade gliomas.

	AUC (95% confidence interval)	Significance (*P*-value)	Cut-off	Sensitivity (%)	Specificity (%)
ASL-nCBF model	0.888 (0.783–0.993)	<0.0001	0.517	85.7	85.7
DSC-nrCBF model	0.962 (0.913–1.000)	<0.0001	0.556	89.3	92.9

AUC, area under the curve; ASL-nCBF, arterial spin labeling normalized cerebral blood flow; DSC-nrCBF, dynamic susceptibility contrast normalized relative cerebral blood flow.

The performance of the radiomics-based classification models was further evaluated using the test set (*n* = 4). Both the ASL-nCBF and DSC-nrCBF models correctly predicted the class (LGG or HGG) of all patients included in the test set (Tables 2 and 3). Furthermore, the cut-off values determined from the ROC analysis could also correctly differentiate the LGG and HGG patients.

**Table 2:** Prediction results of the ASL-nCBF classification model for low- and high-grade gliomas.

Test data # (*n* = 4)	#1 (Diffuse astrocytoma, grade II)	#2 (Anaplastic oligodendroglioma, grade III)	#3 (Glioblastoma, grade IV)	#4 (Glioblastoma, grade IV)
logit(*p*)_ASL-nCBF_^a^	−0.411	0.673	1.897	1.371
Probability (*p*)^b^	0.399	0.662	0.870	0.797
Predicted class	LGG	HGG	HGG	HGG

^a^Cut-off value: 0.517; ^b^*p*: probability of a patient with high-grade glioma (0 < *p* < 1); ASL-nCBF, arterial spin labeling normalized cerebral blood flow; LGG, low-grade glioma; HGG, high-grade glioma.

**Table 3:** Prediction results of the DSC-nrCBF classification model for low- and high-grade gliomas.

Test data # (*n* = 4)	#1 (Diffuse astrocytoma, grade II)	#2 (Anaplastic oligodendroglioma, grade III)	#3 (Glioblastoma, grade IV)	#4 (Glioblastoma, grade IV)
logit(*p*)_DSC-nrCBF_^a^	−2.379	0.829	5.154	1.106
Probability (*p*)^b^	0.085	0.696	0.994	0.751
Predicted class	LGG	HGG	HGG	HGG

^a^Cut-off value: 0.556; ^b^*p*: probability of a patient with high-grade glioma (0 < *p* < 1); DSC-nrCBF, dynamic susceptibility contrast normalized relative cerebral blood flow; LGG, low-grade glioma; HGG, high-grade glioma.

## Discussion

This study compared 91 radiomic features in gliomas between ASL-nCBF and DSC-nrCBF. To our knowledge, this is the first study focusing on the comparison between ASL- and DSC-derived perfusion parameters in gliomas using radiomics. Compared with DSC imaging, ASL imaging underestimates CBF and better characterizes perfusion heterogeneity. However, there are a great number of radiomic features with positive correlations between ASL-nCBF and DSC-nrCBF. Moreover, the radiomics-based classification models showed high diagnostic performance for differentiating between LGGs and HGGs.

The main first-order statistics, including the mean, median, 90^th^ percentile, and maximum values of ASL-nCBF tended to be lower than those of DSC-nrCBF. This could be explained from the fact that ASL tends to underestimate CBF due to increased MTT (e.g., complex and heterogeneous vasculature in tumors or low blood flow in white matter regions)^11,13^. Our ASL-CBF and DSC-rCBF maps were normalized to normal-appearing white matter (NAWM). Previous studies also reported that ASL-CBF tends to be lower than DSC-rCBF when normalized to NAWM^11,12^. Additionally, DSC imaging tends to overestimate rCBF because of its high sensitivity to large vessels^25^. In this study, large vessels were excluded from the tumor volume by using a semi-automated segmentation method. However, intratumoral microvessels might not have been removed completely. This could explain the hyperperfusion observed in DSC imaging, particularly in the maximum values.

The second- or higher-order statistics has been used for the assessment of texture pattern in brain tumors^23,24,26^. The texture analysis in our study included the GLCM-, GLDM-, GLRLM-, GLSZM-, and neighboring gray-tone difference matrix (NGTDM)-based features. The 82.2% texture features that were significantly different (*P* < 0.00055) between ASL-nCBF and DSC-nrCBF mainly point that ASL-nCBF is more heterogeneous (GLCM-based Idm, GLCM-based Id, GLDM-based gray-level variance, GLDM-based large dependence emphasis, and GLRLM-based run entropy) with greater variations in neighboring voxel values (GLCM-based joint entropy and GLCM-based sum entropy) than DSC-nrCBF. Furthermore, the GLDM-based high gray-level emphasis and GLRLM-based high gray-level run emphasis suggest that ASL-nCBF consists of a greater proportion of high voxel values in the segmented tumor region, while the GLDM-based low gray-level emphasis, GLRLM-based low gray-level run emphasis, and GLSZM-based low gray-level zone emphasis suggest that ASL-nCBF consists of a smaller proportion of low voxel values. These findings are consistent with the trend observed in our first-order-based features, such as entropy, uniformity, and skewness.

Intratumoral heterogeneity is an important predictor of tumor prognosis^27^. Preclinical studies in ASL imaging have demonstrated that CBF exhibits intratumoral heterogeneity in rat and mouse glioma models^28,29^. The present study revealed that ASL-nCBF is more heterogeneous than DSC-nrCBF. The discrepancy in perfusion patterns between ASL and DSC imaging may be attributed to their principles and methodology. ASL measures CBF by using labeled arterial water as a freely diffusible tracer, which distributes across capillary membranes throughout the brain tissue^11^ and is not affected by the destruction of the blood–brain barrier (BBB)^10^. On the contrary, DSC imaging utilizes an exogenous contrast agent as a non-diffusible tracer and is sensitive to the BBB destruction^30^. ASL imaging more accurately reflects the physiological perfusion of tissues and can objectively evaluate the degree of angiogenesis and malignancy in brain tumors due to the different diffusivity of the tracers^14,31^. These suggest that ASL-nCBF reflects glioma perfusion heterogeneity better than DSC-nrCBF. Therefore, ASL imaging has potential for characterizing perfusion heterogeneity in gliomas.

MR perfusion-weighted imaging studies have reported that ASL-CBF is positively correlated with DSC-rCBF and DSC-rCBV in brain tumors^11–13^. For the first-order-based mean in this study, there was a strong positive correlation between ASL-nCBF and DSC-nrCBF (*ρ* = 0.73, *P* < 0.00055) in gliomas. This finding agrees with those reported previously^11–13^. Besides the mean, strong and moderate positive correlations were observed in the first-order based median (*ρ* = 0.70, *P* < 0.00055), 90^th^ percentile (*ρ* = 0.75, *P* < 0.00055), and maximum (*ρ* = 0.57, *P* < 0.00055). This suggests that even though ASL imaging underestimates CBF compared with DSC imaging, ASL imaging could be an alternative method to DSC imaging for evaluating glioma perfusion. On the other hand, very strong positive correlations (*r* ≥ 0.8 or *ρ* ≥ 0.8) between ASL-nCBF and DSC-nrCBF were found in texture features. Some features with significant correlations were, for example, the GLCM-based joint entropy and GLCM-based sum entropy, which are associated with randomness of voxel values, and the GLDM-, GLRLM-, GLSZM-based gray-level non-uniformity, and NGTDM-based coarseness, which are associated with heterogeneous texture. These relationships between ASL and DSC imaging imply that both techniques may evaluate similar perfusion patterns (e.g., randomness and heterogeneity) in gliomas.

The ROC analysis showed that the DSC-nrCBF model has better diagnostic performance for differentiating HGGs from LGGs. Several studies have applied radiomics to MR imaging and reported diagnostic performance for distinguishing between LGGs and HGGs^23,24,26^. A study showed that a logistic regression model using radiomic features from ASL-CBF had an AUC, sensitivity, and specificity of 0.750, 71.4%, and 63.9%, respectively^23^. Combining radiomic features from various MR imaging techniques improved the model performance with an AUC, sensitivity, and specificity of 0.911, 85.2%, and 85%, respectively^23^. Another study reported that the AUC, sensitivity, and specificity of a support vector machine model using 30 texture features from multi-parametric MR imaging, including ASL imaging, were 0.987, 96.4%, and 97.3%, respectively^24^. Zacharaki *et al*. proposed a support vector machine model, including DSC-rCBV, with an AUC, sensitivity, and specificity of 0.896, 84.6%, and 95.5%, respectively^26^. Our models have a diagnostic performance comparable to that of these studies for distinguishing between LGGs and HGGs. Furthermore, our ASL-nCBF and DSC-nrCBF models have a relatively high performance, with their AUCs being not significantly different (*P* ≥ 0.05). These imply that preoperative glioma grading using radiomics may be feasible with only a single MR perfusion-weighted imaging-derived parameter, such as CBF. Additionally, most selected features for model construction were related to texture patterns. This suggests that texture analysis in MR perfusion-weighted imaging is useful for distinguishing HGGs from LGGs. The ASL-nCBF model is interchangeable to the DSC-nrCBF model for glioma grading and should be preferred, especially for patients who cannot undergo DSC imaging.

This study has several limitations. First, the sample size was small. To avoid any bias in patient selection, the glioma patients were enrolled consecutively. Even though there was this limitation, it was possible to identify differences and correlations between radiomic features of ASL and DSC imaging-derived parameters, which was the main purpose of our study, and to reach a conclusion about the perfusion patterns of ASL and DSC imaging. A future study with a larger sample size may yield improved ASL and DSC models for glioma grading. Second, post-labeling delay (PLD), one of the scan parameters of ASL imaging, was fixed at 1525 ms for all cases. If the PLD is shorter/longer than the arrival time of labeled arterial blood, CBF may not be assessed correctly. Ideally, PLD is set by considering the patient background (e.g., age and medical history). However, it is difficult to set a PLD for each individual patient with brain tumor. Third, the ASL and DSC imaging data were acquired at different resolutions (voxel spacing; ASL: 1.9 mm × 1.9 mm × 4.0 mm, DSC: 1.7 mm × 1.7 mm × 6.0 mm). This may have contributed to the increased glioma perfusion heterogeneity in ASL imaging and differences in the texture features between ASL and DSC imaging. Despite these limitations, our findings demonstrated that radiomics is useful for characterizing glioma perfusion in ASL and DSC imaging and for classifying LGGs and HGGs.

In conclusion, radiomics in ASL and DSC imaging showed that ASL imaging has lower CBF and reflects glioma perfusion heterogeneity better than DSC imaging, whereas both techniques characterize similar perfusion patterns. For classifying LGGs and HGGs, the DSC-nrCBF model showed higher diagnostic performance and was comparable to the ASL-nCBF model. Radiomics can provide glioma perfusion patterns quantitatively, which can be used to differentiate gliomas, and ASL imaging can be a non-invasive alternative to DSC imaging for evaluating glioma perfusion.

## Materials and methods

### Patient population

This retrospective observational study was approved by our institutional review board (Osaka University Ethics Committee, approval number 17225-2), and passive informed consent was obtained in the form of opt-out on the institutional website from all individual patients. All procedures performed were in accordance with the ethical standards of the institutional review board and with the 1964 Helsinki declaration and its later amendments or comparable ethical standards. Fifty-four patients with pathologically proven gliomas who underwent pseudo-continuous ASL and DSC perfusion-weighted imaging during the same session in our institution prior to any treatment and/or biopsy between January 2014 and December 2019 were eligible for this study. All patients’ histopathological diagnoses fulfilled the 2007 or 2016 WHO classification criteria of the central nervous system tumors^32,33^. The flow diagram for patient selection is shown in Fig. 1. Eight patients were excluded due to the following reasons: (1) inadequate quality of MR images (e.g., motion and susceptibility artifacts) for tumor segmentation (*n* = 4), (2) WHO grade I gliomas (*n* = 3), and (3) pilomyxoid astrocytoma (*n* = 1). WHO grade I gliomas, such as pilocytic astrocytoma, are benign and are considered a separate entity^34^. A definite grading for pilomyxoid astrocytoma is not currently recommended according to the 2016 WHO classification criteria^33^. Finally, 46 glioma patients, including 15 LGGs and 31 HGGs, were enrolled in this study.

### MR imaging

All patients underwent multi-parametric MR imaging using a 3-T scanner (Discovery MR750 3.0 T, GE Healthcare, Milwaukee, WI, USA) with a 32-channel head coil. The following 7 MR imaging sequences were acquired: (1) T_1_ fluid-attenuated inversion recovery (FLAIR) image, (2) T_2_ FLAIR image, (3) T_2_-weighted image (T_2_WI), (4) T_2_^*^-weighted image (T_2_^*^WI), (5) pseudo-continuous ASL imaging, (6) DSC imaging, and (7) contrast-enhanced T_1_-weighted image (CE-T_1_WI). The main parameters of ASL and DSC imaging were set as follows. Axial ASL imaging, acquired using a three-dimensional pseudo-continuous ASL with spiral fast spin-echo sequence: PLD = 1525 ms, repetition time (TR) = 4642 ms, echo time (TE) = 10.5 ms, flip angle (FA) = 111°, 512 sampling points on 8 spiral arms, in-plane matrix = 128 × 128, field of view (FOV) = 240 mm, slice thickness = 4 mm, band width (BW) = ±62.5 kHz, number of slices = 37, scan time = 2 min 1 s. Oblique-axial DSC imaging, acquired using a gradient-echo echo-planar imaging sequence after a bolus injection of 0.1 ml/kg of gadoterate meglumine (MAGNESCOPE, Guerbet, Tokyo, Japan) at a rate of 3 ml/s, followed by a 30 ml bolus of saline flush at the same rate: TR = 2000 ms, TE = 13.3 ms, FA = 60°, matrix size = 96 × 128, FOV = 220 mm, slice thickness = 5 mm, slice spacing = 1 mm, BW = ±250 kHz, number of slices = 22, scan time = 1 min 20 s. Before DSC imaging, a bolus injection of 0.1 ml/kg of gadoterate meglumine was used for dynamic contrast-enhanced imaging (preload leakage correction to minimize T_1_ effects for DSC imaging). The scan parameters of the anatomical MR imaging, T_1_ FLAIR, T_2_ FLAIR, T_2_WI, T_2_^*^WI, and CE-T_1_WI, are described in Supplementary Text S1.

### Post-processing

The post-processing of the ASL and DSC imaging data was performed with 3DASL and BrainStatAIF applications implemented with FuncTool (version 14.3.01, GE Healthcare, Milwaukee, WI, USA), respectively. The ASL-CBF and DSC-rCBF maps for each patient were generated from ASL imaging and DSC imaging, respectively. Motion correction and arterial input function (AIF) deconvolution with singular value decomposition were applied to the DSC-derived map. The AIF locations were detected automatically.

All MR images of each patient were registered to the patient’s own oblique-axial CE-T_1_WI, which was set to align along the anterior commissure–posterior commissure line, using Functional MR Imaging of the Brain Software Library (version 5.0.9, FMRIB Analysis Group, University of Oxford, Oxford, UK). Then, these MR images were resliced to match the oblique-axial CE-T_1_WI.

Additionally, a 6.8-mm diameter spherical volume-of-interest (VOI) (162.8 mm^3^) was placed within NAWM, avoiding any abnormalities, in CE-T_1_WIs and T_2_ FLAIR images. The VOIs were transferred to all perfusion maps in order to normalize ASL-CBF and DSC-rCBF in each patient. The VOI measurements were performed using ITK-SNAP (version 3.6.0, Penn Image Computing and Science Laboratory, University of Pennsylvania, Philadelphia, PA, USA). All voxels in the ASL-CBF and DSC-rCBF maps were divided by the mean value of the NAWM VOI in the respective map, whereby the ASL-nCBF and DSC-nrCBF maps were generated.

### Tumor segmentation

**Figure 5:**
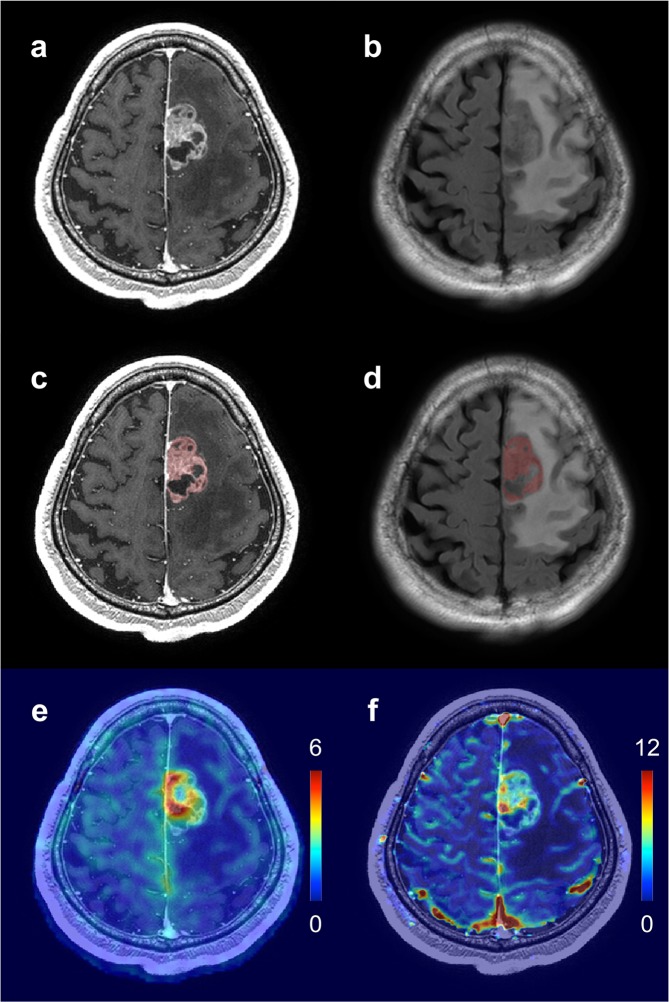
Example of tumor segmentation for a patient with WHO grade IV glioblastoma (59-year-old male). (**a**) CE-T_1_WI. (**b**) T_2_ FLAIR image. (**c**) The segmented tumor volume (*red color*) overlaid on the CE-T_1_WI. (**d**) The segmented tumor volume (*red color*) overlaid on the T_2_ FLAIR image. (**e**) The ASL-nCBF map overlaid on the CE-T_1_WI. (**f**) The DSC-nrCBF map overlaid on the CE-T_1_WI. WHO, World Health Organization; CE-T_1_WI, contrast-enhanced T_1_-weighted image; FLAIR, fluid-attenuated inversion recovery; ASL-nCBF, arterial spin labeling normalized cerebral blood flow; DSC-nrCBF, dynamic susceptibility contrast normalized relative cerebral blood flow.

A supervised random forests machine-learning algorithm implemented with ITK-SNAP was used for semi-automatic tumor segmentation. CE-T_1_WIs and T_2_ FLAIR images were used to cross-reference the solid portion of the tumor in all patients, which was defined as the gadolinium-enhanced region on the CE-T_1_WIs and/or the signal abnormality region on the T_2_ FLAIR images. The machine-learning algorithm took into account the intensities of both MR images during segmentation^35^. T_1_ FLAIR images, T_2_WIs, and T_2_^*^WIs were used to exclude any cysts, calcifications, edema, hemorrhage, necrosis, and large vessels in the tumor volume. Figure 5 shows an example of tumor segmentation.

### Radiomic feature extraction

The segmented tumor volume was used as a mask image for radiomic feature extraction. A total of 91 radiomic features (Supplementary Table 1) were extracted from each of the ASL-nCBF and DSC-nrCBF maps by using PyRadiomics (version 1.3.0, Computational Imaging and Bioinformatics Lab, Harvard Medical School, Boston, MA, USA)^22^. The extracted radiomic features can be divided into the following 6 classes: (1) first-order statistics (18 features), (2) GLCM (22 features), (3) GLDM (14 features), (4) GLRLM (16 features), (5) GLSZM (16 features), and (6) NGTDM (5 features). All radiomic features were extracted using the default parameters of PyRadiomics. The feature descriptions and mathematical definitions can be found elsewhere (http://www.radiomics.io/pyradiomics.html)^22^.

### Statistical analysis

All values are expressed as the mean ± standard deviation. All statistical analyses were conducted using R software (version 3.5.0, R Foundation for Statistical Computing, Vienna, Austria). A *P*-value of < 0.05 was considered statistically significant.

The paired *t*-test or Wilcoxon signed-rank test was used to compare the radiomic features between ASL-nCBF and DSC-nrCBF. Pearson’s product-moment correlation coefficients (*r*) or Spearman’s rank-order correlation coefficients (*ρ*) were used to examine the correlations of the radiomic features between ASL-nCBF and DSC-nrCBF. The Shapiro–Wilk test was used for testing the data normality. The Bonferroni correction method for multiple comparisons was applied to adjust the significance level (*α*), resulting in *P* < 0.00055 (*α* = 0.05/91) to be considered statistically significant.

Before the construction of the radiomics-based classification models, the sample was randomly divided into a training set (*n* = 42) and a test set (*n* = 4). The test set included 1 patient with WHO grade II, 1 patient with WHO grade III, and 2 patients with WHO grade IV gliomas. The LASSO regression was used to select the radiomic features that best classify LGGs and HGGs in the ASL-nCBF and DSC-nrCBF maps (training set, *n* = 42). Leave-one-out cross validation was used for the tuning parameter (*λ*) selection. The radiomic features with non-zero coefficients were selected. The selected features were used to construct a logistic regression model for each perfusion map, as follows:

3 $${\rm{l}}{\rm{o}}{\rm{g}}{\rm{i}}{\rm{t}}(p)=\,{\rm{l}}{\rm{n}}\left(\frac{{p}}{1-{p}}\right)={\beta }_{0}+{\beta }_{1}{x}_{1}+{\beta }_{2}{x}_{2}+\ldots +{\beta }_{i}{x}_{i}$$

where *p* indicates the probability of a patient with HGG (0 < *p* < 1), and *β*_i_ and *x*_i_ indicate regression coefficients and explanatory variables, respectively. In this study, the *x*_i_ variables corresponded to the selected radiomic features.

ROC analysis was performed to assess the performance of each model for differentiating between LGGs and HGGs. The empirical method by DeLong *et al*.^36^ was used to compare the AUCs of the ASL-nCBF and DSC-nrCBF models. Additionally, the test set was used to evaluate whether the models can correctly predict the class (LGG or HGG) of new patients.

## Supplementary information

Supplementary Information.

## Data Availability

All data generated or analyzed during this study are included in this published article and its Supplementary Information files.
