# Supplementary material for: A radiomics-based comparative study on arterial spin labeling and dynamic susceptibility contrast perfusion-weighted imaging in gliomas
Source: Sci Rep. 2020 Apr 9;10:6121. doi: 10.1038/s41598-020-62658-9 (PMC7145821; doi:10.1038/s41598-020-62658-9)
Supplement: Supplementary file 1 — Supplementary Information. [file 41598_2020_62658_MOESM1_ESM.pdf]

Scientific Reports

A radiomics-based comparative study on arterial spin labeling and dynamic susceptibility contrast perfusion-weighted imaging in gliomas

Authors and affiliations

Takashi Hashido[1,2], Shigeyoshi Saito[2], Takayuki Ishida[2,*]

[1] Department of Medical Technology, Osaka University Hospital, Suita, Osaka, Japan

[2] Department of Medical Physics and Engineering, Division of Health Science, Osaka University Graduate School of Medicine, Suita, Osaka, Japan

[*]**Corresponding author information**

Takayuki Ishida, PhD

Department of Medical Physics and Engineering, Division of Health Science, Osaka University Graduate School of Medicine

1-7 Yamadaoka, Suita, Osaka 565-0871, Japan

E-mail address: tishida@sahs.med.osaka-u.ac.jp

Supplementary file contents:

Supplementary Table S1

Radiomic features included in this study. All radiomic features extracted by using PyRadiomics (http://www.radiomics.io/pyradiomics.html).

Supplementary Table S2

Comparisons of all radiomic features between ASL-nCBF and DSC-nrCBF in gliomas.

Supplementary Table S3

Correlation coefficients of all radiomic features between ASL-nCBF and DSC-nrCBF in gliomas.

Supplementary Text S1

The scan parameters of the anatomical MR imaging (T_1 FLAIR, T_2 FLAIR, T_2WI, T_2^*WI, and CE-T_1WI) and MR perfusion-weighted imaging (ASL and DSC imaging).

Supplementary Table S1. Radiomic features included in this study.

Feature #	Radiomic feature	Feature #	Radiomic feature	Feature #	Radiomic feature
	**First-order statistics**	32	Autocorrelation	63	Run-length non-uniformity
1	Interquartile range	33	Sum entropy	64	Short run emphasis
2	Skewness	34	Sum squares	65	Long run high gray-level emphasis
3	Uniformity	35	Cluster prominence	66	Run percentage
4	Median	36	Imc2	67	Long run low gray-level emphasis
5	Energy	37	Imc1	68	Run entropy
6	Robust mean absolute deviation	38	Difference average	69	High gray-level run emphasis
7	Mean absolute deviation	39	Id	70	Run-length non-uniformity normalized
8	Total energy	40	Cluster tendency		**GLSZM**
9	Maximum		**GLDM**	71	Gray-level variance
10	Root mean squared	41	Gray-level variance	72	Zone variance

11	90th percentile	42	High gray-level emphasis	73	Gray-level non-uniformity normalized
12	Minimum	43	Dependence entropy	74	Size-zone non-uniformity normalized
13	Entropy	44	Dependence non-uniformity	75	Size-zone non-uniformity
14	Range	45	Gray-level non-uniformity	76	Gray-level non-uniformity
15	Variance	46	Small dependence emphasis	77	Large area emphasis
16	10th percentile	47	Small dependence high gray-level emphasis	78	Small area high gray-level emphasis
17	Kurtosis	48	Dependence non-uniformity normalized	79	Zone percentage
18	Mean	49	Large dependence emphasis	80	Large area low gray-level emphasis
	**GLCM**	50	Large dependence low gray-level emphasis	81	Large area high gray-level emphasis
19	Joint average	51	Dependence variance	82	High gray-level zone emphasis

20	Joint entropy	52	Large dependence high gray-level emphasis	83	Small area emphasis
21	Cluster shade	53	Small dependence low gray-level emphasis	84	Low gray-level zone emphasis
22	Maximum probability	54	Low gray-level emphasis	85	Zone entropy
23	Idmn		**GLRLM**	86	Small area low gray-level emphasis
24	Joint energy	55	Short run low gray-level emphasis		**NGTDM**
25	Contrast	56	Gray-level variance	87	Coarseness
26	Difference entropy	57	Low gray-level run emphasis	88	Complexity
27	Inverse variance	58	Gray-level non-uniformity normalized	89	Strength
28	Difference variance	59	Run variance	90	Contrast
29	Idn	60	Gray-level non-uniformity	91	Busyness
30	Idm	61	Long run emphasis		
31	Correlation	62	Short run high gray-level emphasis		

GLCM, gray-level co-occurrence matrix; GLDM, gray-level dependence matrix; GLRLM, gray-level run-length matrix; GLSZM, gray-level size-zone matrix; NGTDM, neighboring gray-tone difference matrix.

Supplementary Table S2. Comparisons of radiomic features between ASL-nCBF and DSC-nrCBF in gliomas.

Feature #	Radiomic feature	ASL-nCBF	DSC-nrCBF	*P*-value
	**First-order statistics**			
1	*Interquartile range*	1.10 ± 0.77	2.17 ± 1.95	<0.00001*
2	*Skewness*	0.73 ± 0.67	2.23 ± 1.48	<0.00001*
3	*Uniformity*	0.03 ± 0.02	0.10 ± 0.06	<0.00001*
4	Median	1.91 ± 0.79	2.53 ± 1.96	0.05534
5	*Energy*	$6.68\times10^5 \pm 9.39\times10^5$	$3.14\times10^6 \pm 7.64\times10^6$	0.00003*
6	*Robust mean absolute deviation*	0.46 ± 0.32	0.95 ± 0.86	<0.00001*
7	*Mean absolute deviation*	0.64 ± 0.42	1.51 ± 1.36	<0.00001*
8	*Total energy*	$1.32\times10^5 \pm 1.86\times10^5$	$6.20\times10^5 \pm 15.11\times10^5$	0.00003*
9	*Maximum*	5.30 ± 2.83	24.41 ± 21.77	<0.00001*
10	*Root mean squared*	2.21 ± 0.96	3.76 ± 2.94	<0.00001*
11	*90th percentile*	3.15 ± 1.51	5.64 ± 4.75	<0.00001*
12	*Minimum*	0.49 ± 0.31	0.13 ± 0.26	<0.00001*
13	*Entropy*	5.50 ± 0.90	3.94 ± 1.02	<0.00001*
14	*Range*	4.81 ± 2.95	24.29 ± 21.80	<0.00001*

15	*Variance*	0.90 ± 1.08	7.76 ± 14.79	<0.00001*
16	10th percentile	1.14 ± 0.40	1.11 ± 1.01	0.10877
17	*Kurtosis*	3.85 ± 2.31	14.86 ± 16.69	<0.00001*
18	Mean	2.05 ± 0.85	3.06 ± 2.37	0.00552
	**GLCM**			
19	*Joint average*	32.36 ± 17.79	9.98 ± 7.47	<0.00001*
20	*Joint entropy*	8.45 ± 1.55	6.21 ± 1.85	<0.00001*
21	*Cluster shade*	$6.44\times10^4 \pm 12.61\times10^4$	$1.98\times10^4 \pm 6.48\times10^4$	0.00027*
22	*Maximum probability*	0.03 ± 0.02	0.10 ± 0.09	<0.00001*
23	Idmn	0.999 ± 0.001	0.999 ± 0.001	0.72908
24	*Joint energy*	0.008 ± 0.008	0.046 ± 0.045	<0.00001*
25	Contrast	11.47 ± 18.93	7.32 ± 15.62	0.00097
26	*Difference entropy*	2.42 ± 0.63	1.91 ± 0.72	<0.00001*
27	Inverse variance	0.38 ± 0.07	0.38 ± 0.07	0.93102
28	Difference variance	6.11 ± 12.31	4.47 ± 10.09	0.08433
29	Idn	0.98 ± 0.01	0.98 ± 0.01	0.00329
30	*Idm*	0.46 ± 0.12	0.62 ± 0.16	<0.00001*
31	*Correlation*	0.98 ± 0.01	0.94 ± 0.03	<0.00001*

32	*Autocorrelation*	1713.07 ± 1899.91	225.61 ± 358.59	<0.00001*
33	*Sum entropy*	6.52 ± 0.93	4.85 ± 1.10	<0.00001*
34	*Sum squares*	361.79 ± 438.46	75.08 ± 145.66	<0.00001*
35	*Cluster prominence*	$1.71\times10^7 \pm 3.37\times10^7$	$3.88\times10^6 \pm 17.01\times10^6$	<0.00001*
36	*Imc2*	0.995 ± 0.003	0.975 ± 0.015	<0.00001*
37	*Imc1*	-0.47 ± 0.04	-0.43 ± 0.06	<0.00001*
38	*Difference average*	1.96 ± 1.11	1.25 ± 1.09	<0.00001*
39	*Id*	0.52 ± 0.10	0.65 ± 0.14	<0.00001*
40	*Cluster tendency*	1435.70 ± 1740.02	293.02 ± 568.12	<0.00001*
	**GLDM**			
41	*Gray-level variance*	361.06 ± 434.61	75.80 ± 144.24	<0.00001*
42	*High gray-level emphasis*	1687.60 ± 1845.51	226.83 ± 359.59	<0.00001*
43	*Dependence entropy*	8.87 ± 0.61	7.83 ± 0.69	<0.00001*
44	*Dependence non-uniformity*	$1.12\times10^4 \pm 1.08\times10^4$	$7.84\times10^3 \pm 9.43\times10^3$	<0.00001*
45	*Gray-level non-uniformity*	$3.48\times10^3 \pm 4.63\times10^3$	$1.19\times10^4 \pm 1.67\times10^4$	<0.00001*

46	*Small dependence emphasis*	0.10 ± 0.08	0.06 ± 0.07	<0.00001*
47	*Small dependence high gray-level emphasis*	406.45 ± 670.45	74.13 ± 171.40	<0.00001*
48	*Dependence non-uniformity normalized*	0.10 ± 0.03	0.07 ± 0.03	<0.00001*
49	*Large dependence emphasis*	67.83 ± 41.89	167.08 ± 105.55	<0.00001*
50	*Large dependence low gray-level emphasis*	0.85 ± 1.66	28.71 ± 44.12	<0.00001*
51	*Dependence variance*	15.98 ± 7.28	30.66 ± 14.71	<0.00001*
52	*Large dependence high gray-level emphasis*	$4.37\times10^{4} \pm 2.51\times10^{4}$	$6.80\times10^{3} \pm 4.90\times10^{3}$	<0.00001*
53	*Small dependence low gray-level emphasis*	0.0002 ± 0.0002	0.0014 ± 0.0011	<0.00001*
54	*Low gray-level emphasis*	0.006 ± 0.008	0.089 ± 0.090	<0.00001*

	**GLRLM**			
55	*Short run low gray-level emphasis*	0.004 ± 0.005	0.037 ± 0.028	<0.00001*
56	*Gray-level variance*	366.51 ± 438.02	84.00 ± 151.24	<0.00001*
57	*Low gray-level run emphasis*	0.006 ± 0.007	0.071 ± 0.063	<0.00001*
58	*Gray-level non-uniformity normalized*	0.030 ± 0.018	0.085 ± 0.046	<0.00001*
59	*Run variance*	0.78 ± 0.56	2.74 ± 3.37	<0.00001*
60	*Gray-level non-uniformity*	$2.29\times10^3 \pm 2.57\times10^3$	$4.70\times10^3 \pm 4.78\times10^3$	<0.00001*
61	*Long run emphasis*	2.58 ± 1.10	6.27 ± 5.73	<0.00001*
62	*Short run high gray-level emphasis*	1633.12 ± 1825.10	234.99 ± 372.38	<0.00001*
63	*Run-length non-uniformity*	$6.48\times10^4 \pm 6.19\times10^4$	$3.85\times10^4 \pm 4.90\times10^4$	<0.00001*
64	*Short run emphasis*	0.85 ± 0.07	0.72 ± 0.13	<0.00001*

65	*Long run high gray-level emphasis*	2844.38 ± 2401.29	432.40 ± 457.83	<0.00001*
66	*Run percentage*	0.78 ± 0.09	0.62 ± 0.16	<0.00001*
67	*Long run low gray-level emphasis*	0.02 ± 0.05	1.28 ± 3.47	<0.00001*
68	*Run entropy*	6.45 ± 0.63	5.60 ± 0.48	<0.00001*
69	*High gray-level run emphasis*	1762.47 ± 1901.62	254.54 ± 386.05	<0.00001*
70	*Run-length non-uniformity normalized*	0.70 ± 0.11	0.52 ± 0.18	<0.00001*
	**GLSZM**			
71	*Gray-level variance*	406.94 ± 463.55	164.94 ± 216.52	<0.00001*
72	*Zone variance*	$2.56\times10^5 \pm 9.27\times10^5$	$1.09\times10^6 \pm 2.21\times10^6$	<0.00001*
73	Gray-level non-uniformity normalized	0.030 ± 0.022	0.040 ± 0.022	0.00071

74	Size-zone non-uniformity normalized	0.21 ± 0.10	0.21 ± 0.10	0.62609
75	Size-zone non-uniformity	$3.03\times10^{3} \pm 5.49\times10^{3}$	$2.80\times10^{3} \pm 7.80\times10^{3}$	0.01895
76	Gray-level non-uniformity	148.43 ± 149.26	146.66 ± 284.66	0.03409
77	*Large area emphasis*	$2.63\times10^{5} \pm 9.44\times10^{5}$	$1.11\times10^{6} \pm 2.22\times10^{6}$	<0.00001*
78	*Small area high gray-level emphasis*	1475.35 ± 1808.91	402.97 ± 539.05	<0.00001*
79	*Zone percentage*	0.093 ± 0.094	0.055 ± 0.079	0.00009*
80	*Large area low gray-level emphasis*	$5.16\times10^{3} \pm 30.44\times10^{3}$	$2.88\times10^{5} \pm 12.59\times10^{5}$	<0.00001*
81	Large area high gray-level emphasis	$6.24\times10^{7} \pm 18.48\times10^{7}$	$1.65\times10^{7} \pm 2.84\times10^{7}$	0.01781
82	*High gray-level zone emphasis*	2540.97 ± 2599.68	616.43 ± 702.17	<0.00001*
83	Small area emphasis	0.44 ± 0.13	0.44 ± 0.15	0.96862

84	*Low gray-level zone emphasis*	0.008 ± 0.015	0.054 ± 0.054	<0.00001*
85	*Zone entropy*	8.35 ± 0.97	7.69 ± 0.93	<0.00001*
86	*Small area low gray-level emphasis*	0.002 ± 0.004	0.016 ± 0.018	<0.00001*
	**NGTDM**			
87	*Coarseness*	0.0021 ± 0.0036	0.0011 ± 0.0019	<0.00001*
88	Complexity	$4.99\times10^3 \pm 8.05\times10^3$	$5.41\times10^3 \pm 16.02\times10^3$	0.19550
89	Strength	10.61 ± 17.07	6.02 ± 11.97	0.12697
90	*Contrast*	0.038 ± 0.030	0.012 ± 0.011	<0.00001*
91	*Busyness*	1.19 ± 2.65	9.63 ± 14.72	<0.00001*

All values are expressed as the mean ± standard deviation.

*The *italicized radiomic features* had significant differences (paired *t*-test or Wilcoxon signed-rank test, $P < 0.00055$: Bonferroni correction, $\alpha = 0.05/91$) between ASL-nCBF and DSC-nrCBF.

ASL-nCBF, arterial spin labeling normalized cerebral blood flow; DSC-nrCBF, dynamic susceptibility contrast normalized relative cerebral blood flow; GLCM, gray-level co-occurrence matrix; GLDM, gray-level dependence matrix; GLRLM, gray-level run-length matrix; GLSZM, gray-level size-zone matrix; NGTDM, neighboring gray-tone difference matrix.

Supplementary Table S3. Correlation coefficients of radiomic features between ASL-nCBF and DSC-nrCBF.

Feature #	Radiomic feature	Correlation coefficient	*P*-value
44	*GLDM Dependence non-uniformity*	$\rho = 0.97$	<0.00001*
87	*NGTDM Coarseness*	$\rho = 0.97$	<0.00001*
60	*GLRLM Gray-level non-uniformity*	$\rho = 0.95$	<0.00001*
45	*GLDM Gray-level non-uniformity*	$\rho = 0.93$	<0.00001*
63	*GLRLM Run-length non-uniformity*	$\rho = 0.91$	<0.00001*
5	*First-order Energy*	$\rho = 0.87$	<0.00001*
8	*First-order Total energy*	$\rho = 0.87$	<0.00001*
76	*GLSZM Gray-level non-uniformity*	$\rho = 0.82$	<0.00001*
13	*First-order Entropy*	$\rho = 0.82$	<0.00001*
20	*GLCM Joint entropy*	$\rho = 0.81$	<0.00001*
58	*GLRLM Gray-level non-uniformity normalized*	$\rho = 0.81$	<0.00001*
3	*First-order Uniformity*	$\rho = 0.80$	<0.00001*
72	*GLSZM Zone variance*	$\rho = 0.80$	<0.00001*
77	*GLSZM Large area emphasis*	$\rho = 0.79$	<0.00001*
1	*First-order Interquartile range*	$\rho = 0.79$	<0.00001*
85	*GLSZM Zone entropy*	$\rho = 0.79$	<0.00001*

24	*GLCM Joint energy*	$\rho = 0.78$	<0.00001*
6	*First-order Robust mean absolute deviation*	$\rho = 0.78$	<0.00001*
80	*GLSZM Large area low gray-level emphasis*	$\rho = 0.78$	<0.00001*
7	*First-order Mean absolute deviation*	$\rho = 0.77$	<0.00001*
91	*NGTDM Busyness*	$\rho = 0.77$	<0.00001*
33	*GLCM Sum entropy*	$r = 0.81$	<0.00001*
68	*GLRLM Run entropy*	$r = 0.77$	<0.00001*
43	*GLDM Dependence entropy*	$r = 0.76$	<0.00001*
66	*GLRLM Run percentage*	$r = 0.73$	<0.00001*
32	*GLCM Autocorrelation*	$\rho = 0.76$	<0.00001*
70	*GLRLM Run-length non-uniformity normalized*	$r = 0.72$	<0.00001*
11	*First-order 90th percentile*	$\rho = 0.75$	<0.00001*
39	*GLCM Id*	$\rho = 0.75$	<0.00001*
30	*GLCM Idm*	$\rho = 0.75$	<0.00001*
59	*GLRLM Run variance*	$\rho = 0.75$	<0.00001*
42	*GLDM High gray-level emphasis*	$\rho = 0.75$	<0.00001*
61	*GLRLM Long run emphasis*	$\rho = 0.74$	<0.00001*
69	*GLRLM High gray-level run emphasis*	$\rho = 0.74$	<0.00001*

19	*GLCM Joint average*	$\rho = 0.74$	<0.00001*
64	*GLRLM Short run emphasis*	$\rho = 0.74$	<0.00001*
34	*GLCM Sum squares*	$\rho = 0.74$	<0.00001*
40	*GLCM Cluster tendency*	$\rho = 0.73$	<0.00001*
49	*GLDM Large dependence emphasis*	$\rho = 0.73$	<0.00001*
10	*First-order Root mean squared*	$\rho = 0.73$	<0.00001*
18	*First-order Mean*	$\rho = 0.73$	<0.00001*
62	*GLRLM Short run high gray-level emphasis*	$\rho = 0.73$	<0.00001*
75	*GLSZM Size-zone non-uniformity*	$\rho = 0.73$	<0.00001*
41	*GLDM Gray-level variance*	$\rho = 0.72$	<0.00001*
15	*First-order Variance*	$\rho = 0.72$	<0.00001*
4	*First-order Median*	$\rho = 0.70$	<0.00001*
38	*GLCM Difference average*	$\rho = 0.70$	<0.00001*
26	*GLCM Difference entropy*	$\rho = 0.70$	<0.00001*
56	*GLRLM Gray-level variance*	$\rho = 0.69$	<0.00001*
47	*GLDM Small dependence high gray-level emphasis*	$\rho = 0.69$	<0.00001*
46	*GLDM Small dependence emphasis*	$\rho = 0.69$	<0.00001*
81	*GLSZM Large area high gray-level emphasis*	$\rho = 0.67$	<0.00001*

65	*GLRLM Long run high gray-level emphasis*	$\rho = 0.67$	<0.00001*
35	*GLCM Cluster prominence*	$\rho = 0.64$	<0.00001*
36	*GLCM Imc2*	$\rho = 0.64$	<0.00001*
79	*GLSZM Zone percentage*	$\rho = 0.63$	<0.00001*
90	*NGTDM Contrast*	$\rho = 0.63$	<0.00001*
82	*GLSZM High gray-level zone emphasis*	$\rho = 0.63$	0.00001*
23	*GLCM Idmn*	$\rho = 0.62$	0.00001*
84	*GLSZM Low gray-level zone emphasis*	$\rho = 0.62$	0.00001*
31	*GLCM Correlation*	$\rho = 0.62$	0.00001*
22	*GLCM Maximum probability*	$\rho = 0.61$	0.00001*
14	*First-order Range*	$\rho = 0.61$	0.00001*
73	*GLSZM Gray-level non-uniformity normalized*	$\rho = 0.60$	0.00001*
25	*GLCM Contrast*	$\rho = 0.60$	0.00001*
83	*GLSZM Small area emphasis*	$r = 0.59$	0.00002*
88	*NGTDM Complexity*	$\rho = 0.60$	0.00002*
78	*GLSZM Small area high gray-level emphasis*	$\rho = 0.59$	0.00002*
28	*GLCM Difference variance*	$\rho = 0.57$	0.00004*
21	*GLCM Cluster shade*	$\rho = 0.57$	0.00005*

9	*First-order Maximum*	$\rho = 0.57$	0.00005*
71	*GLSZM Gray-level variance*	$\rho = 0.54$	0.00013*
51	*GLDM Dependence variance*	$r = 0.53$	0.00014*
48	*GLDM Dependence non-uniformity normalized*	$\rho = 0.51$	0.00031*
29	*GLCM Idn*	$\rho = 0.50$	0.00054*
67	GLRLM Long run low gray-level emphasis	$\rho = 0.49$	0.00070
16	First-order 10th percentile	$\rho = 0.48$	0.00079
57	GLRLM Low gray-level run emphasis	$\rho = 0.47$	0.00110
74	GLSZM Size-zone non-uniformity normalized	$\rho = 0.47$	0.00123
50	GLDM Large dependence low gray-level emphasis	$\rho = 0.46$	0.00153
55	GLRLM Short run low gray-level emphasis	$\rho = 0.44$	0.00220
37	GLCM Imc1	$r = 0.42$	0.00411
54	GLDM Low gray-level emphasis	$\rho = 0.42$	0.00427
52	GLDM Large dependence high gray-level emphasis	$\rho = 0.41$	0.00547
89	NGTDM Strength	$\rho = 0.32$	0.03022
86	GLSZM Small area low gray-level emphasis	$\rho = 0.28$	0.06056
12	First-order Minimum	$\rho = 0.18$	0.23732
17	First-order Kurtosis	$\rho = 0.11$	0.47225

27	GLCM Inverse variance	ρ = -0.09	0.53565
53	GLDM Small dependence low gray-level emphasis	ρ = -0.07	0.62542
2	First-order Skewness	ρ = 0.07	0.62774

The radiomic features (Table rows) are arranged in order from the feature with the highest significant correlation, and this order corresponds to that in Fig. 3.

*The *italicized radiomic features* had significant correlations (Pearson's product-moment correlation or Spearman's rank-order correlation, $P < 0.00055$: Bonferroni correction, $\alpha = 0.05/91$) between ASL-nCBF and DSC-nrCBF.

ASL-nCBF, arterial spin labeling normalized cerebral blood flow; DSC-nrCBF, dynamic susceptibility contrast normalized relative cerebral blood flow; GLCM, gray-level co-occurrence matrix; GLDM, gray-level dependence matrix; GLRLM, gray-level run-length matrix; GLSZM, gray-level size-zone matrix; NGTDM, neighboring gray-tone difference matrix; r, Pearson's product-moment correlation coefficient; ρ, Spearman's rank-order correlation coefficient.

Supplementary Text S1. The scan parameters of the anatomical MR imaging (T_1 FLAIR, T_2 FLAIR, T_2WI, T_2^*WI, and CE-T_1WI) and MR perfusion-weighted imaging (ASL and DSC imaging).

All patients underwent multi-parametric magnetic resonance (MR) imaging using a 3-T scanner (Discovery MR750 3.0T, GE Healthcare, Milwaukee, WI, USA) with a 32-channel head coil. In this study, the following 7 MR imaging sequences were acquired: 1) T_1 fluid-attenuated inversion recovery (FLAIR) image, 2) T_2 FLAIR image, 3) T_2-weighted image (T_2WI), 4) T_2^*-weighted image (T_2^*WI), 5) pseudo-continuous arterial spin labeling (ASL) imaging, 6) dynamic susceptibility contrast (DSC) imaging, and 7) contrast-enhanced T_1-weighted image (CE-T_1WI). The main parameters of each sequence were set as follows:

1) Oblique-axial T_1 FLAIR: repetition time (TR) = 2600 ms, echo time (TE) = 24 ms, inversion time (TI) = 1000 ms, flip angle (FA) = 111°, matrix size = 352 × 256, field of view (FOV) = 220 mm, slice thickness = 5 mm, slice spacing = 1 mm, band width (BW) = ± 31.25 kHz, number of slices = 24, scan time = 1 min 57 s.

2) Oblique-axial T_2 FLAIR: TR = 8800 ms, TE = 140 ms, TI = 2041 ms, FA = 111°, matrix size = 320 × 224, FOV = 220 mm, slice thickness = 5 mm, slice spacing = 1 mm, BW = ± 35.71 kHz, number of slices = 24, scan time = 2 min 22 s.

3) Oblique-axial T_2WI: TR = 5800 ms, TE = 96 ms, FA = 111°, matrix size = 512 × 320, FOV = 220 mm, slice thickness = 5 mm, slice spacing = 1 mm, BW = ± 41.67 kHz, number of slices = 24, scan time = 2 min 2 s.

4) Oblique-axial T_2^*WI: TR = 770 ms, TE = 18 ms, FA = 20°, matrix size = 480 × 224, FOV = 220 mm, slice thickness = 5 mm, slice spacing = 1 mm, BW = ± 31.25 kHz, number of slices = 24, scan time = 2 min 55 s.

5) Axial ASL imaging, acquired using a three-dimensional pseudo-continuous ASL with spiral fast spin-echo sequence: PLD = 1525 ms, TR = 4642 ms, TE = 10.5 ms, FA = 111°, 512 sampling points on 8 spiral arms, in-plane matrix = 128 × 128, FOV = 240 mm, slice thickness = 4 mm, BW = ± 62.5 kHz, number of slices = 37, scan time = 2 min 1 s.

6) Oblique-axial DSC imaging, acquired using a gradient-echo echo-planar imaging sequence after a bolus injection of 0.1 ml/kg of gadoterate meglumine (MAGNESCOPE, Guerbet, Tokyo, Japan) at a rate of 3 ml/s, followed by a 30 ml bolus of saline flush at the same rate: TR = 2000 ms, TE = 13.3 ms, FA = 60°, matrix size = 96 × 128, FOV = 220 mm, slice thickness = 5 mm, slice spacing = 1 mm, BW = ± 250 kHz, number of slices = 22, scan time = 1 min 20 s. Before DSC imaging, a bolus injection of 0.1 ml/kg of gadoterate

meglumine was used for dynamic contrast-enhanced imaging (preload leakage correction to minimize T_1 effects for DSC imaging).

7) Oblique-axial CE-T_1WI, acquired using a three-dimensional spoiled gradient-echo sequence after DSC imaging: TR = 8.8 ms, TE = 2.8 ms, FA = 15°, matrix size = 512 × 320, FOV = 240 mm, slice thickness = 0.9 mm, BW = ± 41.67 kHz, number of slices = 210, scan time = 4 min 57 s.

The oblique-axial planes of T_1 FLAIR, T_2 FLAIR, T_2WI, T_2^*WI, DSC imaging, and CE-T_1WI were set to align along the anterior commissure–posterior commissure line of each patient.
